# Research progress in traditional Chinese medicine in the treatment of Alzheimer’s disease and related dementias

**DOI:** 10.3389/fphar.2022.921794

**Published:** 2022-11-24

**Authors:** Wanying Tan, Lingjun Qi, Xiaoyu Hu, Zhenghuai Tan

**Affiliations:** ^1^ Hospital of Chengdu University of Traditional Chinese Medicine, Chengdu, China; ^2^ Sichuan Academy of Traditional Chinese Medicine, Chengdu, China

**Keywords:** Alzheimer’s disease, traditional Chinese medicine, ginseng, *Pueraria lobata*, epimedium

## Abstract

Alzheimer’s disease (AD) is the world’s leading cause of dementia and has become a huge economic burden on nations and families. However, the exact etiology of AD is still unknown, and there are no efficient medicines or methods to prevent the deterioration of cognition. Traditional Chinese medicine (TCM) has made important contributions in the battle against AD based on the characteristics of multiple targets of TCM. This study reviewed the treatment strategies and new discoveries of traditional Chinese medicine in current research, which may be beneficial to new drug researchers.

## Introduction

In 2018, Alzheimer’s Disease International estimated a dementia prevalence of approximately 50 million cases worldwide, which is projected to triple in 2050. The most recent data estimate that the dementia prevalence in Europe will double by 2050 (Scheltens et al., 2021).

The cardinal features of Alzheimer’s disease (AD) pathology are amyloid plaques and neurofibrillary tangles (NFTs). In addition, neuropil threads, dystrophic neurites, and associated astrogliosis and microglial activation are seen, and cerebral amyloid angiopathy frequently coexists. The consequences of these pathological processes include neurodegeneration with synaptic and neuronal loss, leading to macroscopic atrophy (Lane et al., 2018).

The cause of AD is currently unknown; however, many hypotheses exist that attempt to explain the disease etiology. The amyloid hypothesis, the prevalent theory of AD etiologies, suggests that the accumulation of pathological forms of β-amyloid (Aβ), produced by sequential cleavage of amyloid precursor protein (APP) by β- and γ-secretase, in the brain is the primary pathological process, driven by an imbalance between Aβ production and Aβ clearance. The formation of NFTs and the subsequent neuronal dysfunction and degeneration, perhaps mediated by inflammation, are thought to be downstream processes. While fibrillar amyloid within dense-core plaques was originally thought to be critical for the development of AD, it is now thought that soluble Aβ oligomers may be the most pathological forms: oligomers purified from AD brains and applied to neurons *in vitro* inhibit long-term potentiation, cause synaptic dysfunction, damage dendritic spines and cause neuronal death. Human oligomers also induce the hyperphosphorylation of tau at AD-relevant epitopes and cause neuritic dystrophy in cultured neurons. Plaques may therefore act as a “reservoir” from which amyloid oligomers diffuse or may even act as a protective mechanism sequestering toxic Aβ oligomers until they reach a physiological saturation point (Lane et al., 2018).

The central hypothesis is the amyloid cascade hypothesis, which proposes that the deposition of Aβ is the central initiating event and driving force, with Aβ accumulation causing the resulting downstream processes seen in AD (Robinson et al., 2018)

In June 2021, the Food and Drug Administration (FDA) approved the monoclonal antibody aducanumab as an immunotherapeutic approach against AD; aducanumab targets Aβ oligomers and fibrils and is able to reduce Aβ accumulation and slow the progression of cognitive impairment. Aducanumab was also claimed to have an effect on another hallmark of AD, decreasing the level of phosphorylated tau (p-tau) evaluated in cerebrospinal fluid (CSF) by positron emission tomography (PET) (Silvestro et al., 2022). However, the use of aducanumab has created controversy among healthcare professionals due to its association with Alzheimer’s-related imaging abnormalities (ARIAs) and hypersensitivity (Behl et al., 2022). In addition, sodium oligomannate (GV-971) is a marine algae-derived oral oligosaccharide being developed by Shanghai Green Valley Pharmaceuticals for the treatment of AD. Sodium oligomannate received its first approval in November 2019 in China for the treatment of mild to moderate AD to improve cognitive function. However, both aducanumab and sodium oligomannate have little effect on improving cognitive function (Syed, 2020).

Cerebrovascular changes also represent a common risk factor for AD. Vascular remodeling and pathological changes to the macro- and microvasculature may disrupt blood vessel integrity. Notably, such remodeling leads to vascular disease and cerebral hypoperfusion associated with neuronal injury, as well as structural and functional brain damage. Moreover, hemodynamic impairment of the mean blood flow velocity, pulsatility index, and cerebrovascular reactivity were found in AD patients compared with controls using transcranial Doppler to monitor the middle cerebral artery. These results suggest hemodynamic impairment as a critical marker of cognitive decline and confirm its role as an early predictor of vascular damage in AD (Raz et al., 2016). A variety of cerebrovascular diseases, such as cerebral hemorrhage, cerebral infarction, and subarachnoid hemorrhage, can cause dementia, including vascular dementia (VD). Cerebral blood vessels play a role in brain health, not only for the delivery of oxygen and nutrients but also for the trophic signaling that inextricably links the wellbeing of neurons and glia to that of cerebrovascular cells (Iadecola, 2013; Xing et al., 2014). Often coexisting with AD, mixed VD and neurodegenerative dementia have emerged as the leading cause of age-related cognitive impairment.

The cholinergic system has been suggested as the earliest and most affected system associated with AD pathophysiology. Moreover, cholinesterase inhibitors (ChEIs) are a potential class of drugs that can amplify cholinergic activity to improve cognition and global performance and reduce psychiatric and behavioral disturbances (Jabir et al., 2018).

Currently, only four drugs are approved by the FDA for the treatment of AD. Three of these are inhibitors of the enzyme acetylcholinesterase (AChE) (donepezil, galantamine and rivastigmine), and one is an N-methyl-D-aspartate (NMDA) receptor antagonist (memantine). However, these drugs contribute to only modest benefits in symptom management. Furthermore, these drugs do not prevent neuronal loss, brain atrophy or, consequently, the progressive deterioration of cognition (Vaz and Silvestre, 2020).

In China, traditional Chinese medicine (TCM) often classifies AD into the categories of “forgetfulness”, “consumptive”, “amnesia” or “dementia”. “Dementia” was first described as an independent disease during the Ming Dynasty in the book *Jingyue Quanshu miscellaneous syndrome*. TCM treatment of AD is mostly based on the identification of patterns, such as *deficiency* syndrome, and the driving away of pathogens or purgation and tonification. According to the pathogenesis characteristics, “kidney deficiency with marrow depletion is root causes, turbid phlegm accumulation is symptoms, five viscera disorder, brain marrow dysfunction”, doctors of past dynasties used a variety of effective treatments, such as tonifying the kidney (*Shen*) and replenishing marrow (*Sui*), strengthening the spleen (Pi) and benefiting vital *Qi*, eliminating phlegm (Tan) for resuscitation, promoting blood circulation and invigorating *Q*i (Liu et al., 2012). TCM has multiple targets; therefore, it has certain advantages in the prevention and treatment of AD.

The current literature review aims to summarize the recent advances in the involvement of TCM prescriptions, Chinese materia medica, and active compounds in Alzheimer’s disease and related dementias ameliorating effects and underlying mechanisms in clinical or preclinical studies by consulting PubMed, China National Knowledge Infrastructure, and China Science and Technology Journal. We also review its phytochemistry and its explanation in TCM, which may help to understand pharmacological activities.

## TCM prescription

### Dian Kuang Meng Xing decoction

Wang Qingren, a famous physician in the Qing Dynasty, first created Dian Kuang Meng Xing decoction (DKMXD). DKMXD contains many TCMs, such as Radix Paeoniae Rubra, Semen Persicae, Radix Bupleurum, Rhizoma Cyperi, Pericarpium Citri Reticulatae, Pinellia Ternata, Cortex Mori, Fructus Perillae, Pericarpium Arecae, Akebiae Caulis, and Radix Glycyrrhizae. The use of DKMXD for dialectical treatment has been shown to improve the daily activity ability and neurological ability of AD patients. Among 50 AD patients, DKMXD was significantly effective in 48 patients and was effective in two patients (Kong and Sun, 2009).

In a clinical observational study involving 42 patients with AD, DKMXD effectively improved the therapeutic effect, quality of life, and cognitive function of patients (Guo, 2019).

In a study that used DKMXD or Piracetam to treat VD for 28 days, the results showed that the Mini-Mental State Examination (MMSE) score and related biochemical test results were better in the DKMXD group than in the Piracetam group (Cheng et al., 2014).

### Huanglian Wendan decoction

Huanglian Wendan decoction (HLWDD) comes from the “*Liu Yin Tiao Bian*.” HLWDD regulates qi and dissipates phlegm. HLWDD is composed of rhizoma pinelliae, pericarpium citri reticulatae, poria, radix astragali, semen cassia, ruizoma coptidis, caulis bambusae in taenie, radix puerariae, radix et rhizoma salviae miltiorrhizae, radix glycyrrhizae and other Chinese herbs.

Some studies have suggested that HLWDD can have a therapeutic effect on VD model rats by inhibiting the mRNA expression levels of interleukin-1 beta (IL-1β), tumor necrosis factor-α (TNF-α) and cyclooxygenase-2 (COX-2). Furthermore, it can improve the activities of catalase (CAT), glutathione peroxidase (GSH-Px) and superoxide dismutase (SOD) and reduce the activity of malondialdehyde (MDA). HLWDD significantly improved learning and memory impairment and pathological changes in the hippocampus in VD model rats (Dou et al., 2019; Dou et al., 2021). Zheng Wanli et al. used HLWDD combined with acupuncture in the treatment of 60 patients with VD and Western medicine (nimodipine (dihydropyridine calcium antagonists), Naofukang (Piracetam), and Dukexi (Almitrine and Raubasine tablets) as a control in the treatment of 60 additional patients. After treatment, the MMSE score of the HLWDD + acupuncture treatment group was significantly higher than that of the Western medicine control group (Zhen and Liu, 2006).

### Tongqiao Huoxue decoction

Tongqiao Huoxue decoction (TQHXD) comes from “*Yi Lin Gai Cuo*” in the Qing Dynasty. TQHXD is mainly composed of Radix Paeoniae Rubra, Ligusticum chuanxiong, semen persicae, Flos Carthami, Moschus and other drugs. TQHXD has the effect of activating blood, dispelling stasis, and dredging collaterals.

In a study of VD patients taking donepezil hydrochloride or TQHXD for 3 months, the cognitive ability and daily living ability of the patients were significantly improved, the MMSE and activities of daily living (ADL) scores in the two groups were significantly improved, and the improvement in the TQHXD group was better than that in the donepezil group. The regional cerebral blood flow (CBF) in the TQHXD group was significantly higher than that before treatment or in the donepezil group. The plasma homocysteine level in the TQHXD group was significantly lower than that in the donepezil group (Zhu et al., 2019).

In a study of 92 patients with VD, the routine treatment group received Western medicine treatment (Oxiracetam), and the combination treatment group received the routine treatment plus TQHXD and acupuncture. TQHXD and acupuncture combined with Western medicine treatment had significant curative effects on patients with VD, including the upregulation of serum levels of acetylcholine (Ach), serotonin, norepinephrine, dopamine (DA) and other neurotransmitters and significant improvements in mental state and daily life abilities (Fang, 2021).The study found that TQHXD combined with acupuncture had significant effects on AD patients with Qi stagnation and blood stasis syndrome, including significant relief of clinical symptoms, improved cognitive function, and reduced serum homocysteine levels (Cui, 2021). In a clinical observational study of 122 patients with poststroke cognitive impairment (Li J. et al., 2019a), TQHXD combined with “Huiyang Nine Needles” improved cognitive function and daily living abilities and had anti-inflammatory, antioxidant and blood rheological effects, which were beneficial for promoting the recovery of cognitive function. Jin et al. (2021) found that TQHXD combined with butylphthalide had good safety and a significant curative effect on chronic cerebral ischemia in elderly individuals, could improve clinical symptoms and the cerebral hypoperfusion state, and protected cognitive function, which was related to the regulation of high mobility group box protein B1 (HMGB1), soluble intercellular adhesion molecule 1 (sICAM-1), chemokine ligand 12 (CXCL12), and lipoprotein-associated phospholipase A2 (Lp-PLA2) expression.

TQHXD reduced the rate of neuronal loss and improved neuroplasticity, synaptic remodeling, and learning and memory in rats with VD (Wei et al., 2018). TQHXD could significantly improve the learning ability of VD rats by reducing the calcium ion concentration in hippocampal neurons and improving the neurons which has been impaired by elevated intracellular calcium ions (Ge et al., 2015a).TQHXD could significantly improve the learning of VD rats by reducing the calcium ion concentration in hippocampal neurons and damaging abnormally impaired neurons, which elevated intracellular calcium ions in nerve cells and memory ability (Ge et al., 2015a). In addition, TQHXD could also increase the content of choline acetyltransferase (ChAT) and reduce the content of acetylcholinesterase (AChE) in the hippocampus, improve hemorheological indicators, and improve learning and memory ability in VD rats (Ge et al., 2015b; Wang et al., 2015).

### Yi Qi Cong Ming decoction

“*Yi Fang Ji Jie*” recorded the use of Yi Qi Cong Ming decoction (YQCMD), which consisted of Radix Astragali, ginseng, Radix Pueraria, Fructose Viticis, Radix Paeoniae Alba, Cortex Phellodendri, Rhizoma Cimicifugae and roasted licorice. YQCMD has been widely used in neurological diseases such as senile dementia (AD and VD). YQCMD was shown to regulate the content of DA, serotonin, and norepinephrine in the cerebral cortex of VD rats, thereby improving learning and memory ability (Peng et al., 2018); reducing the expression of IL-6, TNF-α, and IL-1β; and inhibiting the inflammatory response in the hippocampus of rats with VD (Li W. et al., 2019).

### Huanglian Jiedu decoction

Huanglian Jiedu decoction (HLJDD) comes from “*Wai Tai Mi Yao*” and functions to increase blood lipids, lower blood pressure, regulate blood sugar, prevent atherosclerosis, and protect brain neurons. HLJDD consists of four herbs, namely, Coptidis rhizome, Scutellariae radix, Phellodendri chinensis cortex, and Gardeniae fructus. The clinical efficacy of HLJDD in the treatment of AD and other forms of dementia has appeared in many clinical trials (Shao et al., 2012; Liu, 2016; Wang, 2021). The pharmacological research results of HLJDD for senile dementia showed that it could play a role in anti-inflammation as well as in inhibiting the production of Aβ and the hyperphosphorylation of tau protein, antioxidant activity, free radical scavenging activity, regulating neurotransmitter expression, and regulating immune activity (Durairajan et al., 2017; Sun et al., 2017). In terms of inhibiting Aβ, intervention with HLJDD in APP/PS1 transgenic AD model mice reduced the mRNA expression of β-amyloid precursor protein (β-APP) in brain tissue (Feng, 2015). HLJDD reduced the destruction of hippocampal nerve cells and the formation of senile plaques in APP/PS1 mice. HLJDD could increase the levels of Ach in brain tissue and the levels of norepinephrine (NE), 5-hydroxytryptamine (5-HT) and other neurotransmitters in the serum of AD mice induced by chronic restraint stress stimulation combined with D-galactose (D-gal) (Qiu et al., 2011b). In terms of brain neuroprotection, nerve growth factor (NGF) provides nutritional support for and contributes to the growth of cholinergic neurons. Insulin-like growth factor-I (IGF-I) provides nutritional protection for brain neurons, contributes to the extension of synapses and improves the survival rate of neurons. HLJDD was shown to upregulate the mRNA expression levels of NGF and IGF-I in the cerebral cortex and hippocampus of SAM-P/8 AD model mice (Peng et al., 2012).

In terms of anti-inflammatory and immune-regulating activity, HLJDD also has an inhibitory effect on the inflammatory state of the central nervous system (CNS) in the pathological process of AD (Du et al., 2015). HLJDD can downregulate the level of IL-6 in the brain tissue of APP/PS1 transgenic AD model mice (Qiu et al., 2011a). In a rat model in which AD was induced by intracerebroventricular injection of Aβ_1-42_, the expression of nuclear factor κB (an inhibitor of NF-κB, I-κB) in the hippocampus decreased and showed a proinflammatory state. HLJDD upregulated the expression of I-κB and downregulated NF-κB expression in the hippocampus of AD model rats, inhibiting the inflammatory state (Dong et al., 2012). HLJDD improved learning and memory disorders in Aβ_1-42_-treated AD mice, and the underlying mechanisms may involve NMDA receptor-mediated glutamatergic transmission and the adenosine/ATP/AMPK cascade (Liu Y. et al., 2019). In a study in which AD was induced in rats by Aβ25–35A injection, it was found that HLJDD significantly improved learning and memory impairment, decreased Aβ25–35 and APP levels in the hippocampus, increased SOD and GSH activities, reduced MDA concentrations and alleviated nuclear and cytoplasmic abnormalities in the hippocampal CA1 region (Wu et al., 2020).

### Twenty-five flavor coral pills

Twenty-five flavor coral pills are composed of 25 Tibetan medicinal materials, such as coral, pearl, concha margaritifera, Fructus chebulae, Flos carthami, Pyrethrum tatsienense, Acorus calamus L., moschus and Radix aucklandiae. These pills are used to treat various forms of neuropathic pain, such as “white vein disease” and confusion. The pills have the effects of inducing resuscitation, dredging collaterals and relieving pain.

Clinical studies have evaluated TCM syndromes, simple mental state scales and Hasegawa dementia scale scores in patients with vascular cognitive impairment and have indicated that twenty-five flavor coral pills have a good therapeutic effect on these factors. A study found that in a mouse model of AD induced by D-gal combined with aluminum trichloride, the content of p-tau protein in the hippocampus was significantly reduced after treatment with twenty-five flavor coral pills. In the hippocampus and serum, the levels of SOD, CAT, and T-AOC were significantly increased, and the level of MDA was significantly decreased. The protein expression levels of p-mTOR and p-Akt in the hippocampus were significantly increased, and the protein expression of GSK-3β was significantly decreased. Twenty-five flavor coral pills improved the decline in learning and memory ability and oxidative damage, and the mechanism may be related to the Akt/mTOR/GSK-3β signaling pathway (Luo et al.). In mice with scopolamine-induced learning and memory impairment, twenty-five flavor coral pills could increase the expression of neuron-specific nucleoprotein (NeuN) in the cortex and have a protective effect on scopolamine-induced learning and memory impairment in mice. The mechanism may be that these pills play an antioxidant role by regulating the Kelch-like epichlorohydrin related protein 1 (Keap1)/Nrf2/HO-1 signaling pathway (Zhang B. Y. et al., 2022).

### Danggui Shaoyao powder

Danggui Shaoyao San (DSS) is from “*Jinguiyaolue (Synopsis of Prescriptions of the Golden Chamber)*,” which is composed of six Chinese herbs: Radix Angelicae Sinensis, Chinese herbaceous peony, Rhizoma Chuanxiong, Rhizoma Atractylodis, Rhizoma Alismatis and Poria cocos. Studies have shown that DSS has an effect on free-radical-mediated neurological diseases, has anti-inflammatory and antioxidant activities, and reduces hippocampal apoptosis; in addition, DSS mediates the regulation of the central monoamine neurotransmitter system, improving the dysfunction of the central cholinergic nervous system and the scopolamine-induced decrease in Ach levels. DSS can improve the function of dopaminergic, adrenergic, 5-hydroxytryptamine and other systems (Fu et al., 2016).

DSS can improve the movement disorder and cognitive function decline of AD patients (Itoh et al., 1996). JD-30 is an active fraction extracted from Danggui-Shaoyao-San (DSS), a traditional Chinese medicinal prescription. Long-term treatment with JD-30 significantly decreased the prolonged latency of SAMP8 cells in the Morris water maze test. It also ameliorated the reduction of long-term potentiation (LTP) and reduced the damage of neurons in the hippocampus, the content and deposition of Aβ in the brain of SAMP8 (Hu et al., 2012), increased the number of cholinergic-positive nerve fibers in the hippocampal dentate gyrus and the number of hippocampal synapses, improved the structure of postsynaptic terminals (He et al., 2008), inhibited the deposition of Aβ in the hippocampus and neuroinflammation, and improved learning and memory in various animal models of dementia. DSS can improve learning and memory ability in rat models of VD and may exert its therapeutic role through the PI3K/AKT signaling pathway (Li X. et al., 2019), regulating the contents of the hippocampal neuropeptides calcitonin gene-related peptide (CGRP), endothelin 1 (ET-1) and somatostatin (SS) (Li S. et al., 2010).

In the APP/PS1 mouse model, DSS alleviated cognitive deficits and improved neuronal degeneration. DSS reduced the deposition of amyloidosis and amyloid β1-42 in the brains of APP/PS1 mice, downregulated the receptor for advanced glycation end products (RAGE), and upregulated the level of low-density lipoprotein receptor-related protein-1. However, in the Aβ protein production pathway, the expression of β-APP, β-secretase and presenilin-1 (PS1) and the expression of insulin-degrading enzymes did not change. It has been shown that DSS can improve amyloidosis and neuronal degeneration in AD, which may be related to its upregulation of lipoprotein receptor-related protein-1 and downregulation of RAGE. (Yang et al., 2021). Biochemical measurements showed that compared with wild-type mice, APP/PS1 mice had elevated triglyceride (TG), total cholesterol (TC), low-density lipoprotein cholesterol (LDL-c), and high-density lipoprotein cholesterol (HDL-c) levels that were decreased, and DSS significantly delayed these changes. Low docosahexaenoic acid (DHA) content and low calcium-independent phospholipase A2 (iPLA2) and 15-LOX expression were observed in the hippocampus and cortex of APP/PS1 mice, and DSS extract significantly reversed these changes. In addition, APP/PS1 mice had abnormal SOD and reactive oxygen species (ROS) levels and decreased MDA and GSH levels, while DSS extract significantly alleviated these changes. In addition, DSS reduced the levels of prostaglandin E2 (PEG2), thromboxane B2 (TXB2), and leukotriene B4 (LTB4) and attenuated the expression of cytoplasmic phospholipase A2 (cPLA2), cyclooxygenase (COX)-1, and COX-2 in the hippocampus and cortex of APP/PS1 mice. DSS plays a positive and effective role in increasing DHA content by upregulating iPLA2 and lipoxygenase, thereby improving oxidative stress and inflammation in APP/PS1 mice and ultimately improving cognitive deficits (Huang et al., 2020).

DSS protected against lipopolysaccharide (LPS)-induced neuroinflammation in BV-2 microglia through the Toll-like receptor (TLR)/NF-κB signaling pathway (Ding et al., 2018).

### Buyang Huanwu decoction

Buyang Huanwu decoction (BHD) comes from the famous doctor Wang Qingren’s “*Yi Lin Gai Cuo*” in the Qing Dynasty. BHD is composed of Astragalus membranaceus; *Angelica sinensis* tail; radix paeoniae rubra, the root of common peony; the rhizome of chuanxiong; earthworm; peach kernel and safflower (*Carthamus*). BHD has the effects of tonifying Qi, activating blood circulation and dredging collaterals and is mainly used for cerebrovascular diseases. At present, an increasing number of basic research and clinical research studies have found that BHD can effectively improve learning and memory ability and cognitive and behavioral function. Studies have found that BHD can inhibit the inflammatory response of brain tissue and reduce neuronal apoptosis in AD model mice, and researchers speculate that this neuroprotective effect may be related to the regulation of the inflammatory p38MAPK/NF-κB pathway (Dong et al., 2020). Fei et al. (2014) used APP/PS1 transgenic AD model mice as research subjects and found that after feeding with medium and high doses of BHD, the APP content in the brains was significantly reduced, and the time to find a platform in the Morris water maze (MWM) test was shortened. After removing the platform, the number of stays in the target quadrant area increased, suggesting that the expression of β-APP in the hippocampus was downregulated, the content of Aβ was reduced, and the formation of senile plaques was inhibited. BHD alleviates senile plaque damage to neurons and cognitive impairment by regulating the inflammatory p38MAPK/NF-κB pathway in APP/PS1 AD mice. BHD can also inhibit the expression of the β-hydrolase gene and reduce the generation of Aβ by APP protein, thereby achieving the effect of treating AD.

In AD patient brains, high levels of both soluble Aβ oligomers and insoluble Aβ fibers can activate microglia. Activated microglia release a large number of inflammatory mediators, such as IL-1β, IL-6 and TNF-α, by activating signaling pathways such as nuclear transcription factor (NFκB), mitogen-activated protein kinase (MAPK) and protein kinase C (PKC). High levels of IL-1β in the brain can activate astrocytes, further amplify the inflammatory response, and induce neuronal death. Some studies have found that BHD can block the initiation of the inflammatory response; reduce the expression of the inflammatory factors IL-1β, TNF-α and IL-1 by reducing the content of NF-κB in the brains of AD transgenic mice; inhibit the CNS; restrain the nervous system inflammatory response; and reduce cognitive and memory impairment in model mice (Yu et al., 2017). In addition, animal experiments have shown that BHD can increase the expression of low-density lipoprotein receptor-related protein-1 (LRP1) and reduce the content of RAGE. BHD can significantly alleviate the damage to synaptic transmission and gene expression in the hippocampus induced by ischemia and improve cognitive functions (Li W. et al., 2010a; Tang et al., 2013; Ding et al., 2017). BHD can improve synaptic plasticity, nerve recovery, nerve cell growth and synaptic regeneration in rats with cerebral ischemia (Guo et al., 2015; Pan et al., 2017; Zhou et al., 2018). In addition, BHD promotes angiogenesis after cerebral ischemia–reperfusion injury by targeting the SIRT1/vascular endothelial growth factor (VEGF) pathway and reduces the infiltration of natural killer cells (Zheng et al., 2018). After BHD treatment of VD, the levels of vascular endothelial NO and PGI2, brain-derived neurotrophic factor (BDNF) and NGF *in vivo*, and other factors related to improving perfusion, antioxidant effects, and nerve repair were changed. It was considered that BHD can improve VD (Huang et al., 2015; Chou et al., 2019). A total of 106 patients with VD were treated by electroacupuncture combined with BHD, and this combined treatment significantly improved the symptoms; increased the blood flow velocity of the middle cerebral artery; and increased the serum BDNF, VEGF, and B-cell lymphoma-2 (BCL-2) levels. The combined treatment decreased Bcl-2-related X protein (Bax), cysteine protease (Caspase)-3 and other apoptosis-related factors (Wang et al.).

Some studies aimed to explore the protective mechanism of BHD on neurovascular units through the RAGE/LRP1 pathway in an AD cell model. BHD regulated Aβ metabolism through the RAGE/LRP1 pathway, inhibited the vascular endothelial inflammation induced by intercellular cell adhesion molecule-1 (ICAM-1) and vascular cell adhesion molecule-1 (VCAM-1), and improved the morphological changes caused by Aβ-induced brain microvascular endothelial cell injury. (Liu W. et al., 2019). See Table 1 for summary.

**TABLE 1:** Treatment of Alzheimer’s disease with TCM prescription.

Herbal formula	Ingredients	Possible mechanism	Major findings	Ref.
*Dian Kuang Meng Xing decoction*	Radix Paeoniae Rubra, Semen Persicae, Radix Bupleurum, Rhizoma Cyperi, Pericarpium Citri Reticulatae, Pinellia Ternata, Cortex Mori, Fructus Perillae, Pericarpium Arecae, Akebiae Caulis, and Radix Glycyrrhizae		Improve the daily activity ability, neurological ability, quality of life, cognitive function and the MMSE score of patients in AD	Kong and Sun (2009), Liu et al. (2012), Guo (2019)
*Huanglian Wendan decoction*	rhizoma pinelliae, pericarpium citri reticulatae, poria, radix astragali, semen cassia, ruizoma coptidis, caulis bambusae in taenie, radix puerariae, radix et rhizoma salviae miltiorrhizae, radix glycyrrhizae	Anti-inflammatory and inhibiting IL-1β, TNF-α and COX-2; Antioxidant and improve the activities of CAT, GSH-Px and SOD, and reduce the level of MDA	Improve the MMSE score; Improve the learning and memory impairment	Cheng et al. (2014), Dou et al. (2019), Dou et al. (2021)
*Tongqiao Huoxue decoction*	Radix Paeoniae Rubra, Ligusticum chuanxiong, semen persicae, Flos Carthami, Moschus	Anti-inflammatory, antioxidant and improve the blood rheological effects; Reduce neuronal loss, improved neuro pl asticity and synaptic remodeling. Reduce the activity of AChE and increase the acitivity of ChAT and the levels of Ach, serotonin, and DA	Improve the cognitive ability, daily living ability, learning and memory ability, the MMSE and ADL	Zhen and Liu, (2006), Zhu et al. (2019), Cui, (2021), Fang, (2021), Wang et al. (2015), Ge et al. (2015b)
*Yi Qi Cong Ming decoction*	Radix Astragali, ginseng, Radix Pueraria, Fructus Viticis, Radix Paeoniae Alba, Cortex Phellodendri, Rhizoma Cimicifugae and roasted licorice	Regulate the content of DA, serotonin, and norepinephrine; Inhibiting the expression of IL-6, TNF-α, IL-1β and the inflammatory response in the hippocampus	Improve learning and memory ability	Peng et al. (2018), Li W. et al. (2019)
*Huanglian Jiedu decoction*	Coptidis rhizome, Scutellariae radix, Phellodendri chinensis cortex, and Gardeniae fructus	Anti-inflammation--downregulate the level of IL-6, NF-κB, and antioxidant; Inhibit the production of Aβ and the hyperphosphorylation of tau protein and prevented the degeneration of hippocampal nerve cells; Increase the levels of Ach, NE, 5-HT, Upregulate NGF and IGF-I in the cerebral cortex and hippocampus	Improve learning and memory disorders	Sun et al. (2017), Durairajan et al. (2017), Qiu et al. (2011b), Qiu et al. (2011a), Dong et al. (2012), Wu et al. (2020)
*Twenty-five flavor coral pills*	coral, pearl, concha margaritifera, Fructus chebulae, Flos carthami, Pyrethrum tatsienense, Acorus calamus L., moschus and Radix aucklandiae	Reduce p-tau protein, MDA; Increased the levels of SOD, CAT, and T-AOC; Reduce neuron loss	Improve the decline in learning and memory ability; have a protective effect on scopolamine-induced learning and memory impairment	Luo et al., Bo-Yu Zhang et al. (2022)
*Danggui Shaoyao powder*	Radix Angelicae Sinensis, Chinese herbaceous peony, Rhizoma Chuanxiong, Rhizoma Atractylodis, Rhizoma Alismatis and Poria cocos	Improve the central cholinergic nervous system, improve the function of dopaminergic, adrenergic, 5-HT; Decrease the deposition of Aβ, regulate the expression of the neuropeptides CGRP, ET-1 and SS in hippocampa, Anti-inflammatory, antioxidant activities	Improve learning and memory ability, alleviated cognitive deficits	Fu et al. (2016), Itoh et al. (1996), He et al. (2008), Li S. et al. (2010), Li X. et al. (2019)
*Buyang Huanwu decoction*	astragalus membranaceus; *Angelica sinensis* tail; radix paeoniae rubra, the root of common peony; the rhizome of chuanxiong; earthworm; peach kernel and safflower (*Carthamus*)	Inhibit the inflammatory response: reduce the expression of IL-1β, TNF-α and IL-1; inhibited the vascular endothelial inflammation and reduce neuronal apoptosis; Reduced the levels of APP, β-APP, and Aβ, inhibited the formation of senile plaques; Improve nerve recovery and growth, and synaptic regeneration and plasticity	Improve learning and memory ability and cognitive and behavioral function	Jiang, (2010), Fei et al. (2014), Yu et al. (2017), Dong et al. (2020)

## Single TCMs and their active ingredients

In recent years, many scholars have adopted modern extraction technologies to extract active ingredients or parts from TCMs, such as huperzine A (HupA) from *Huperzia serrata*, curcumin from turmeric, *cistanche deserticola* polysaccharide (CDPS) from *Cistanche deserticola*, ginseng polysaccharide and its components from ginseng, longan ginseng polysaccharide from Longan ginseng, *Morinda officinalis* oligosaccharide from *Morinda officinalis*, icariin from Epimedium, and Osteolipin B and dihydroflavone methyl ether of *Psoralea corylifolia* from *Psoralea corylifolia*. All of these extracts have good curative effects on all AD patient symptoms as well as the pathological characteristics of AD pathogenesis. See Table 2 for summary.

**TABLE 2:** Treatment of Alzheimer’s disease with single TCMs and their active ingredients.

Herbal formula	Main active ingredients	Possible mechanism	Major findings	Reference
*Salvia miltiorrhiza*	Diterpenoid quinones; hydrophilic phenolic acids; Danshensu; Tanshinones Salvianolic acid	Downregulating the expression of β-secretase and increasing the activity of α-secretase and decrease the production of Aβ, inhibition of abnormal phosphorylation of tau protein; Antioxidant, antiapoptotic and anti-inflammatory effects and AChE inhibitory activity; inhibit the proliferation of astrocytes	Improve learning and memory function	Chou et al. (2014), Bae et al. (2019), Chong et al. (2019), Li Z. et al. (2021)
*Ginkgo biloba*	Terpene lactones (bilobalide and ginkgolides A, B, and C); flavone glycosides (isorhamnetin, quercetin, and kaempferol)	Dilating cerebral blood vessels, increasing CBF, and preventing thrombosis; Increase the expression of synaptophysin in the hippocampus; Reduce the loss of neurons and apical dendrites in the hippocampal CA1 area and increase synaptic connections	Enhance the spatial learning and memory ability; Improved cognitive function, Neuropsychiatric symptoms, ADLs and quality of life in patients with mild to moderate dementia	Huang et al. (2004), Zhang et al. (2008), Tan et al. (2015), Xu et al. (2020)
*Fructus Corni*	Fructus Corni iridoid glycoside; iridoid glycoside (Loganin)	Antiapoptotic, anti-inflammatory; Reducing Aβ content and tau hyperphosphorylation; Increasing neurotrophic factors	Improving the learning and memory ability and cognitive impairment	Elsabagh et al. (2005), Gauthier and Schlaefke, (2014), Ma et al. (2021a)
*Epimedium*	Icariin Icariside II	Anti-neurotoxicity, anti-inflammatory and reduced COX-2 and TNF-α; Antioxidant; Inhibit the activity of γ-secretase and downregulate the expression of the PS1 gene; inhibits the expression of APP and BACE-1 and decrease the production of Aβ1-42	Improving learning and memory	Jin et al. (2019), Sheng et al. (2017), Zeng et al. (2010), Li et al. (2022b)
*Turmeric*	Curcumin	Inhibits the formation of Aβ plaques and promotes their breakdown, attenuates tau hyperphosphorylation and enhances tau clearance. Binds copper, lowers cholesterol, modulates microglial activity, inhibits AChE, modulates insulin signaling and acts an antioxidant; reduces apoptosis, neuroinflammation, and glutamate neurotoxicity, exerted neuroprotective effects	Improves neurological and behavioral deficits; Improve the learning and memory ability	Tang and Taghibiglou (2017), Pluta et al. (2022), Han et al. (2018), Wang et al. (2014)
*Pueraria lobata*		Neuroprotective effect; Reduce the level of Aβ and tau protein hyperphosphorylation; Decrease the levels of BDNF, AChE and GSK3β in the hippocampus and cerebral cortex.	Improve cognition and memory.	Li et al. (2017a), Mei et al. (2016), Wu et al. (2017), Zhao et al. (2015)
*Ginseng*	Ginsenosides; Gintonin	Antioxidant; Inhibiting Aβ aggregation and tau hyperphosphorylation, preventing neuroinflammation and apoptosis, increasing neurotrophic factor secretion, and improving mitochondrial dysfunction; Increase the levels of gamma-aminobutyric acid, Ach, and DA, and decrease the levels of glutamate and aspartate in the hippocampus and cerebral cortex	Improved learning and memory	Razgonova et al. (2019), Li et al. (2014), Chu et al. (2014), Cao et al. (2016)
*Dendrobium*	Dendrobium nobile total alkaloids Dendrobium officinale polysaccharide	Decrease the level of amyloid plaques and tau protein hyperphosphorylation, Inhibiting neuroinflammation and neuronal apoptosis, activating autophagy, and enhancing synaptic connections; protecting synaptic integrity; reduce the loss of neurons and Nissl bodies in the hippocampus and cortex	Improve cognitive dysfunction, improving learning and memory	Zhang et al. (2016a), Li et al. (2022a), Feng et al. (2019)
*Coptis chinensis*	Berberine	Reducing ER stress and oxidative stress; Reducing Aβ production and tau levels	Improve spatial learning and memory retention, improved cognitive behavior in the MWM test	Chen et al. (2020a), Xuan et al. (2020), Ye et al. (2021), Hussien et al. (2018), Liang et al. (2021)
*Ligusticum chuanxiong*	Ligustrazine	Dilates blood vessels, improves cerebral ischemia and protects neurons; Increase the expression of NEP, promote the clearance of Aβ, decrease tau hyperphosphorylation and Aβ accumulation; Inhibit mitochondrial dysfunction and apoptosis	Improve the learning and memory ability	Liu et al. (2016b), Zhang et al. (2021a), Guan et al. (2015)
*Huperzia serrata*	HupA	Combated oxidative damage and mitochondrial dysfunction; Competitively inhibit the activity of AChE and reduce neuronal cell death; Activated α-secretase cleavage and downregulated β/γ-secretase	Improves cognition and task switching ability	Gul et al. (2019), Po et al. (2019)
*Polygonum cuspidatum*	Physion8-O-β-glucopyranoside	Increasing the levels of Ach, 5-HT, NE, and DA in the hippocampus; Reducing the content of Aβ and upregulating the expression of drebrin	Enhance the learning and memory ability	Zhang et al. (2019), Xu et al. (2015)
*Gardenia*	Geniposide	Reducing amyloid plaques, inhibiting tau phosphorylation, preventing memory impairment and synaptic loss. Reducing oxidative stress and chronic inflammatory responses, and promoting neurite outgrowth	Improves cognitive and behavioral abilities	Xiang et al. (2018), Chen et al. (2020b), Zhang et al. (2021b)
*Polygala*		Decrease the levels of Aβ and tau protein; Anti-inflammatory, antioxidant, and antiapoptotic effects	Improve cognitive impairment	Li et al. (2022c), Sun et al. (2013), Wang et al. (2020b)
*Rhodiola*	salidroside (Sal), aglycone (tyrosol), rhodopsin, pyridine	Reduce the deposition of Aβ1-42 and neuroinflammation; Inhibiting pyroptosis, reducing tau phosphorylation, and reducing Aβ accumulation; Reducing the levels of the proinflammatory factors IL-1β, IL-6 and TNF-α	Improve cognitive decline during aging	Cai et al. (2021), Xie et al. (2020b), Wang et al. (2020a)
*Acorus tatarinowii*	β-Asarone	Repair the synaptic structure; Reducing the production of Aβ, the number of senile plaques and autophagosomes in the hippocampus	Improve learning and memory	Zhang et al. (2014), Liu et al. (2016a), Yang et al. (2016), Deng et al. (2020)
*Gastrodia elata*	Gastrodin	Modulation of neurotransmitters and mitochondrial cascades; Neuroprotective activities; Inhibition of microglial activation; Reduce the proteolytic process of APP, amyloid deposits and the activity of AChE	Improved the spatial memory and cognitive deficits	Huang et al. (2013), Liu et al. (2018b), Park et al. (2015), Hu et al. (2014)
*Lycium barbarum*	Lycium barbarum polysaccharide	Reduce oxidative stress, restore mitochondrial function, alleviate DNA damage; reduce Aβ levels	Improve cognitive function, learning and memory deficits	Ni et al. (2021), Zhou et al. (2020b), Liu et al. (2021), Meng et al. (2022)
*Cistanche deserticola*	Acteoside	Restoring the homeostasis of the gut-microbiota-brain axis; Alleviating amino acid imbalance, peripheral inflammation, and oxidative stress; Increase hippocampal neurons and Nissl bodies	Improved cognitive function	Ye et al. (2015), Liu et al. (2018a)

### Salvia miltiorrhiza

Salvia is the dried roots and rhizomes of *Salvia miltiorrhiza* Bge., a Lamiaceae plant, which can promote blood circulation and remove blood stasis and cool and nourish blood. Salvia is often used to treat blood stasis blockage of collaterals. *Salvia miltiorrhiza* has various neuroprotective effects related to AD, such as anti-Aβ, antioxidant, antiapoptotic and anti-inflammatory effects and AChE inhibitory activity. Furthermore, *Salvia miltiorrhiza* promotes the neurogenesis of neural progenitor/stem cells *in vitro* and *in vivo*. (Zhang X. Z. et al., 2016).

A study found that *Salvia miltiorrhiza* could effectively reduce the activity of cholinesterase in the brain and serum and significantly improve learning and memory function in model mice in which AD was induced by AlCl_3_ (Chou et al., 2014). Chemical and pharmacological studies have shown that diterpenoid quinones and hydrophilic phenolic acids are the principal bioactive components in *Salvia miltiorrhiza*, whereas tanshinones are the main hydrophobic components in *Salvia miltiorrhiza*.

Danshensu is one of the most important water-soluble components of *Salvia miltiorrhiza*. Danshensu was shown to have an inhibitory effect on the activity of monoamine oxidase A but no inhibitory effect on monoamine oxidase B. In addition, a Danshensu treatment group showed increased DA, protein kinase A and cyclic adenosine monophosphate response element binding protein levels in the cerebral cortex. The results showed that Danshensu treatment could significantly improve the DA signaling cascade in mice with scopolamine- and Aβ-induced cognitive impairment (Bae et al., 2019).

Tanshinone can improve learning and memory ability, reduce the inflammatory response of brain tissue, regulate apoptosis-related proteins (Bcl-2, Bax, and caspase-3), and inhibit the apoptosis of hippocampal cells in AD rats (Li Z. et al., 2021). Tanshinone has a certain neuroprotective effect.

Tanshinone I is a bioactive lipophilic compound mainly isolated from *Salvia miltiorrhiza*. Tanshinone was reported to have antioxidant and anti-inflammatory effects in an ischemic injury model. Tanshinone I can reduce the formation of Aβ42 fibers and breakdown Aβ42 aggregates. Tanshinone I can upregulate antioxidant enzymes such as Mn superoxide dismutase, glutathione peroxidase, and the catalytic subunit and modified subunit of γ-glutamate cysteine ligase. This finding reveals the ability of tanshinone I to maintain mitochondrial function by increasing the expression of Nrf2 (Chong et al., 2019).

Cryptotanshinone is also a type of tanshinone. Multiple studies have shown that the activity of cryptotanshinone is associated with a reduction in Aβ aggregation and toxicity and an upregulation of α-secretase. Cryptotanshinone reduces memory decline and neuroinflammation in Aβ42-injected mice, supporting the anti-Aβ ability of cryptotanshinone (Maione et al., 2018).

Tan IIA is a fat-soluble component isolated and extracted from *Salvia miltiorrhiza*, and it has a wide range of biological activities. In recent years, studies have found that TanIIA plays an active role in the prevention and treatment of AD, mainly in the inhibition of the formation and aggregation of Aβ, the inhibition of abnormal phosphorylation of tau protein, and the protection of neurons (Li Y. et al., 2017). TanIIA treatment can inhibit the proliferation of astrocytes, reduce the level of NF-κB, and increase NeuN, Nissl bodies and inhibitor of NF-κB (IκB) in an AD rat model, all of which exert anti-inflammatory and neuroprotective effects (Li et al., 2015).

Salvianolic acids A and B are active components in *Salvia miltiorrhiza*, and due to its polyphenolic structure, it shows strong free radical scavenging abilities as well as antiapoptotic and anti-inflammatory properties. SalVA A may prevent Aβ-induced damage by reducing Aβ aggregation. Total salvianolic acids from *Salvia miltiorrhiza* reduced learning and memory impairment in APPswe/PS1dE9 mice by reducing Aβ42 and Aβ40 (Shen et al., 2017). Salvianolic acid B can prevent Aβ-induced cytotoxicity and reduce the amyloidogenic pathway by downregulating the expression of β-secretase and increasing the activity of α-secretase that cleaves APP in the nonamyloidogenic pathway. This activity of salvianolic acid B promotes the development of APP toward nonamyloidogenesis and may provide an alternative approach to reducing Aβ production (Chong et al., 2019).

### Ginkgo biloba

*Ginkgo biloba* is the dried leaves of *Ginkgo biloba* (Ginkgobiloba L.). *Ginkgo biloba* extract (EGb) can be used to treat AD symptoms, including cognitive impairment and psychobehavioral symptoms. Major compounds of *G. biloba* are terpene lactones (bilobalide and ginkgolides A, B, and C) and flavone glycosides (isorhamnetin, quercetin, and kaempferol) (Eisvand et al., 2020). Because EGb has the pharmacological effects of dilating cerebral blood vessels, increasing CBF, and preventing thrombosis, it is more clinically used to treat VD than AD (Xu and Hu, 2019). EGb could improve blood viscosity and viscoelasticity, which may facilitate blood perfusion and effectively increase cerebral blood flow (Huang et al., 2004). EGb can promote the expression of γ-aminobutyric acid transporter-1 (GAT1) and CREB in the hippocampus, increase carotid microvessel density, and inhibit the release of inflammatory factors, thereby improving memory in rats with VD (Xu et al., 2020).

The standardized Ginkgo biloba extract EGb761, containing 22%–27% flavonol glycosides, 5%–7% terpene lactones, and less than 5 ppm ginkgolic acids, is one of the most widely used herbal remedies for dementia and cognitive impairment and remains one of the best evaluated and characterized extracts (Tan et al., 2015). EGb761 is internationally recognized as having a therapeutic effect on senile dementia. Studies have shown that EGb761 can significantly increase the expression of synaptophysin in the hippocampus after cerebral ischemia–reperfusion injury, promote synaptic reconstruction, and reduce VD levels. EGb761 can also reduce the persistent loss of neurons and apical dendrites in the hippocampal CA1 area and increase synaptic connections in VD rats (Zhang et al., 2008). EGb761 can significantly enhance the spatial learning and memory ability of VD rats and simultaneously inhibit the apoptosis of neurons in the hippocampus, and the p38MAPK pathway may be involved (Yang et al., 2013). Compared with placebo, EGb761 significantly improved cognitive function, neuropsychiatric symptoms, ADLs and quality of life in patients with mild to moderate dementia. In patients with mild cognitive impairment (MCI), EGb761 also demonstrated significant symptom improvement compared to placebo. EGb761 is listed by the guidelines of the World Federation of Biological Psychiatric Societies as having the same strength of evidence as AChE inhibitors and NMDA antagonists such as memantine (level 3 recommendation; Level B evidence). Only EGb761 has level B evidence for improving cognition, behavior and ADLs in AD and VD patients (Kandiah et al., 2019). Clinical studies of EGb in the treatment of AD have been conducted since the 1980s; however, the benefit of EGb in AD treatment remains controversial. In 2005, 52 college students were randomized to an EGb group or a placebo group (Elsabagh et al., 2005). Another 40 students received EGb and a placebo in the long-term study. In both experiments, students’ performance was assessed on sustained attention, episodic memory, executive function, and complete emotional rating scales. Sustained attention and pattern recognition memory improved after acute ginkgo treatment, but no effect of ginkgo on other tests was observed. There was also no effect on performance on any cognitive test after 6 weeks of treatment. To determine the clinical efficacy of EGb in the treatment of AD, outpatients received EGb or placebo supplements for 26 weeks. Finally, 513 outpatients, including 270 women and 243 men, were randomized to receive 120 mg or 240 mg of EGb daily or placebo for 26 weeks (Schneider et al., 2005). Assessment strategies used the Alzheimer’s Disease Assessment Scale Cognitive Subscale (ADAScog) and Alzheimer’s Disease Collaborative Study Clinical Global Impression Change (ADCS-CGIC) score change from the baseline examinations. No significant differences between the two groups were observed, and the trial did not demonstrate the efficacy of EGb. However, other studies showed that taking a 240-mg daily dose of Ginkgo biloba extract is effective and safe in the treatment of dementia (Gauthier and Schlaefke, 2014; Hashiguchi et al., 2015; Tan et al., 2015).

### Fructose Corni

Fructose Corni is the dried ripe pulp of *Cornus officinalis* Sieb. et Zucc. Fructose Corni can nourish the liver and kidney and is often used to treat dementia and amnesia due to kidney and marrow deficiency. Fructose Corni polysaccharide can induce the expression of brain-derived neurotrophic factor (BDNF) and B-cell lymphoma-2 (Bcl-2) genes in rats, exert its antiapoptotic effect, and promote the repair and self-protection of hippocampal neurons, thereby improving the learning and memory ability of VD rats (Li et al., 2016).

Fructose Corni iridoid glycoside (CIG), the active ingredient of Fructose Corni, improves exercise capacity; attenuates neuronal loss or demyelination; increases the expression of synaptophysin, postsynaptic density protein 95 (PSD-95) and AMPA receptor subunit 1; increases the level of soluble APPα fragment and a disintegrin and metalloproteinase 10 (ADAM10); and decreases receptor-interacting protein kinase-1 (RIPK1) and mixed lineage kinase domain-like protein (MLKL) levels in the brains of SAMP8 mice (Ma et al., 2021a). P301S transgenic mice, an animal model of tauopathy and AD, exhibit tau pathology and synaptic dysfunction. In this model, CIG improves cognitive impairment and survival rates. The main mechanism of its involvement in the regulation of synaptic dysfunction is to inhibit the activation of the Janus kinase-2 (JAK2)/signal transducer and activator of transcription 1 (STAT1) signaling pathway (Ma et al., 2021b). In APP/PS1/tau triple transgenic AD (3×Tg-AD) model mice, CIG can improve learning and memory impairment by reducing Aβ content and tau hyperphosphorylation and increasing neurotrophic factors in the brain (Yang C. et al., 2020). rTg4510 mice are transgenic mice expressing P301 L mutant tau and have been developed as animal models of tau diseases, including AD. CIG gavage for 3 months reduces hyperactivity in rTg4510 mice, preventing neuronal loss and reducing tau hyperphosphorylation and amygdala aggregation (Ma et al., 2020).

Loganin is an iridoid glycoside extracted from Fructus Corni that has been reported to have anti-inflammatory and memory-enhancing properties. Loganin can significantly alleviate anxious behavior and improve memory impairment in 3xTg-AD mice. Aβ deposition in the hippocampus and cortex of 3xTg-AD mice after loganin treatment is reduced. Loganin can also reduce p-tau protein levels in 3xTg-AD mice (Nie et al., 2021). Furthermore, loganin can attenuate Aβ-induced inflammation in BV-2 microglia in part by inactivating the TLR4/TRAF6/NF-κB (TNF receptor-associated factor 6, TRAF6) axis (Cui et al., 2018).

### Epimedium

Epimedium is the dried leaves of *Epimedium brevicornu* Maxim, Epimedium sagittatum (Sieb. Et Zucc. Maxim), *Epimedium pubescens* Maxim, or *Epimedium koreanum* Nakai. Epimedium is spicy, sweet, and warm, and it invigorates the kidney and strengthens yang, dispels wind and removes dampness. Epimedium flavone is the main effective component of Epimedium, which can play a role in maintaining the normal synaptic structure of neurons by promoting the expression of synaptophysin (SYP) and postsynaptic dension-95 (PSD-95) proteins in the brain of APP transgenic mice (Jin et al., 2008). Total flavone of epimedium (TFE) can improve the learning and memory ability of D-galactose (D-gal)/aluminum chloride (AlCl3) induced dementia rats, reduce the activity of AChE in brain and blood tissues, significantly increase bcl-2 and reduce Bax level. Icariin (ICA) is one of the main effective components in total flavonoids of Epimedium (Jian et al., 2009). ICA is one of its main active components and can prevent nerve injury and protect neurons and mitochondria. Icariin can downregulate the gene expression of PS1 in SAMP8 mice, indicating that it can inhibit the activity of γ-secretase and downregulate the expression of the PS1 gene, thereby preventing and treating AD (Zhang et al., 2012)

ICA can promote the survival, proliferation and migration of neural stem cells *in vitro*; promote the proliferation and differentiation of neural stem cells into neurons; and increase the number of cholinergic neurons *in vivo* (Ma et al., 2021c). ICA blocks Aβ_1-42_ production in animal models of AD and inhibits the expression of APP and beta-site APP lyase 1 (BACE-1). ICA can also prevent neurotoxicity caused by hydrogen peroxide (H_2_O_2_), endoplasmic reticulum (ER) stress, ibotenic acid and homocysteine. In addition, ICA is involved in promoting learning and memory in normal aging animals and disease models (Jin et al., 2019).In an Aβ_1-42_-induced AD rat model, ICA was shown to improve synaptic plasticity and exert neuroprotective effects through the BDNF/tyrosine kinase B (TrkB)/Akt pathway, thereby improving learning and memory (Sheng et al., 2017). ICA can inhibit the hyperphosphorylation of tau by inhibiting the PI3K/Akt-dependent GSK-3β signaling pathway, thereby exerting neuroprotective effects on Aβ25-35-treated PC12 cells (Zeng et al., 2010). The improvement effect of ICA on learning and memory was observed in a permanent bilateral common carotid artery ligation (2-VO) rat model, an aluminum-induced AD rat model, and an APP/PS1Tg mouse model (Li L. R. et al., 2022).

Icariside II (ICS II) is the active ingredient of Epimedium, which has anti-inflammatory, antioxidant and anticancer effects. ICSII significantly reduced the protein expression levels of APP, Aβ1-42 and RAGE, reduced the protein expression levels of the inflammatory factors COX-2 and TNF-α, and increased the protein expression level of the anti-inflammatory factor IL-10 in the hippocampus of APP/PS1 transgenic mice by continuous gavage for 7 months. Aβ combined with RAGE can activate microglia and accelerate the inflammatory response. Therefore, ICS II can reduce the inflammatory response of APP/PS1 mice (Zeng et al., 2017).

### Turmeric

Turmeric is the dried rhizome of the ginger plant *Curcuma longa* L. Curcumin is a phenolic pigment extracted from turmeric, Zedoary turmeric, curcuma, etc. The mechanisms by which curcumin alters AD pathology include the following: curcumin inhibits the formation of Aβ plaques and promotes their breakdown, attenuates tau hyperphosphorylation and enhances tau clearance, binds copper, lowers cholesterol, modulates microglial activity, inhibits AChE, modulates insulin signaling and acts an antioxidant (Tang and Taghibiglou, 2017).

The death and atrophy of pyramidal neurons in the hippocampal CA1 region are considered to be typical changes in ischemic brain neurodegeneration and AD. The accumulation of neurotoxic amyloid and dysfunctional tau protein after cerebral ischemia is one of the potential mechanisms of severe neuronal death. Curcumin can affect aging-related cellular proteins, namely, amyloid and tau protein, preventing their aggregation and insolubilization after ischemia. Curcumin also reduces amyloid and tau protein neurotoxicity by affecting their structure. Studies in animal models of cerebral ischemia have shown that curcumin reduces infarct volume, brain edema, blood–brain barrier (BBB) permeability, apoptosis, neuroinflammation, and glutamate neurotoxicity; inhibits autophagy and oxidative stress; and improves neurological and behavioral deficits. Available data suggest that curcumin may be a new therapeutic substance in regenerative medicine and the treatment of neurodegenerative diseases such as postischemic neurodegeneration (Pluta et al., 2022).

Curcumin can improve learning and memory ability, downregulate the level of HMGB1, and reduce the expression of downstream RAGE and TLRs, which in turn reduces the expression of downstream NF-κB in the hippocampus and alleviates AD symptoms in 4-month-old APP/PS1 AD mice (Han et al., 2018).

Another study showed that curcumin exerted neuroprotective effects and induced autophagy through the PI3K/AKT/MTOR pathway in APP/PS1 double-transgenic mice (Wang et al., 2014).Curcumin worked better when used with other compounds (Lin et al., 2020).

The poor water solubility, limited bioavailability, and inability to cross the BBB of curcumin limit its application in the biological field. To overcome these diagnostic and therapeutic limitations, various nanoformulations have also been considered that can enhance the pharmacokinetic properties of curcumin and other bioactive compounds. Numerous nanocarriers have now shown beneficial properties to deliver curcumin and other nutrient compounds through the BBB to efficiently distribute them into the brain (Shabbir et al., 2020).

However, in two studies conducted in China and the United States, there was no significant difference in changes in cognitive function between the placebo and curcumin groups (Baum et al., 2008; Ringman et al., 2012).

### Pueraria lobata

*Pueraria lobata* is the dried root of the legume Puerarialobata (Willd.) Ohwi. This can improve myocardial contraction, reduce myocardial oxygen consumption, expand coronary and cerebral blood vessels and promote blood circulation. It can improve cognition and memory, which may be related to reducing the levels of Aβ and tau hyperphosphorylation by inhibiting the glycogen synthase kinase (GSK) 3β (GSK3β) signaling pathway in the cerebral cortex in APP/PS1 mice (Mei et al., 2016).Studies have shown that *Pueraria lobata* can attenuate Aβ_1-42_-induced tau hyperphosphorylation in SH-SY5Y cells by inhibiting the expression of GSK-3β and activating the Wnt/β-catenin signaling pathway; therefore, *Pueraria lobata* may have a protective effect against AD (Yao et al., 2017). In Aβ_1-42_-induced AD model mice, *Pueraria lobata* restored BDNF, p-tau, malondialdehyde, AChE, and GSK3β levels in the hippocampus and cerebral cortex to a certain extent. In addition, *Pueraria lobata* restored SOD activity and protected neurons from oxidative stress-induced apoptosis (Wu et al., 2017). In a mouse model of sporadic AD (SAD) induced by intracerebroventricular injection of streptozotocin (STZ), *Pueraria lobata* attenuated learning and memory impairment and inhibited oxidative stress in STZ-induced SAD mice (Zhao et al., 2015). *Pueraria lobata* is neuroprotective against Aβ1-42 toxicity by activating estrogen receptors, and ERβ plays a key role in this process (Li L. et al., 2017).

### Ginseng

Ginseng is the dried root and rhizome of Panax ginseng C. A. Mey. Ginseng is warm, sweet, and slightly bitter and has the effects of invigorating vitality, invigorating the spleen, benefiting the lungs, promoting body fluid, soothing the nerves and improving wisdom. Ginsenosides are the main active compounds found in ginseng. Ginsenosides have unique biological activity and medicinal value, namely, antitumor, anti-inflammatory and antioxidant properties, as well as antiapoptotic properties. Studies in recent years have shown that ginsenosides have some positive role in the prevention and treatment of AD (Razgonova et al., 2019). Many ginsenosides have been reported to have the following actions on pathological processes in AD: 1) inhibiting Aβ aggregation and tau hyperphosphorylation, 2) preventing neuroinflammation and apoptosis, 3) increasing neurotrophic factor secretion, and 4) improving mitochondrial dysfunction (Li J. et al., 2021).

The combined use of ginsenoside Rb1 and Rg1 reduces brain Aβ production by modulating multiple factors, including the NLRP3 inflammasome, TNF-α, oxidative stress, astrocytes, and microglial plasma cell activation, and improves cognitive impairment in SAMP8 mice (Yang Y. et al., 2020). Ginsenoside F1 can reduce the level of phosphorylated CREB, increase the level of BDNF in the hippocampal cortex, reduce Aβ plaques, and improve the memory function of APP/PS1 transgenic AD mice (Han et al., 2019). Ginsenoside Rg1 of ginseng extract is the main component that inhibits BACE activity. Ginsenoside Rg1 can stimulate peroxidase proliferator-activated receptor (PPARγ) in AD model cells. Ginsenoside Rg1 inhibits the expression of BACE1, enhances the activity of NF-κB, and increases NF-κB binding to its corresponding site on the β-secretase 1 (BACE1) promoter, thereby inhibiting the translation and transcription of BACE1 and reducing the Aβ produced by the cleavage of APP by BACE1 (Li et al., 2014). In an AD model (Wistar rats injected with STZ in the lateral ventricle), ginsenoside Rg5 was observed to dose-dependently inhibit AChE and activate ChAT, and it improved learning and memory in rats in the MWM test (Chu et al., 2014).

Studies suggest that ginsenosides exert neuroprotective effects by acting on neurotransmitters in AD. For example, ginsenosides can increase the levels of gamma-aminobutyric acid, Ach, and DA and decrease the levels of glutamate and aspartate in the hippocampus and cerebral cortex. In addition, ginsenosides can increase blood levels of glycine and serotonin (Zhang Y, et al., 2016). Apparently, the substances contained in ginseng can increase the levels of Ach in the brains of AD patients. Therefore, ginseng extract can be used for palliative treatment in the early stage of AD.

In the N2a/APP695 cell model (neuronal cells overexpressing APP), it was observed that ginsenoside Re not only reduced the activity of BACE1 and the expression of BACE1 protein but also did not affect the total levels of β-APP and soluble amyloid precursor protein α (sAPPα), while the reduction in BACE1 expression was associated with the activation of ginsenoside Re by PPARγ (Cao et al., 2016).

A randomized controlled trial including forty patients with AD showed the potential efficacy of a heat-processed form of ginseng on cognitive function and behavioral symptoms in patients with moderately severe AD (Heo et al., 2012). In another randomized study, the Alzheimer’s Disease Assessment Scale (ADAS) and Clinical Dementia Rating (CDR) scores improved significantly after high-dose ginseng treatment. Compared with the control group, the baseline value of MMSE in the ginseng treatment group was improved, but this improvement was not statistically significant (Heo et al., 2008). It has efficacy on frontal lobe function in AD (Heo et al., 2016).

Gintonin, a newly discovered ginseng component containing lysophosphatidic acid, alleviates AD-related cranial neuropathy. Gintonin inhibits Aβ-induced neurotoxicity and activates nonamyloidogenic pathways to reduce Aβ formation and increase the expression of Ach and ChAT in the brain *via* lysophosphatidic acid receptors. Gintonin attenuates brain amyloid plaque deposition and promotes the hippocampal cholinergic system and neurogenesis, thereby improving learning and memory impairment. Gintonin also improves cognitive function in AD patients (Kim et al., 2018).Oral administration of gintonin restores cognitive and motor function in animal models of D-gal-induced brain aging, AD, Huntington’s disease (HD), and Parkinson’s disease (PD). The underlying molecular mechanisms of gintonin-mediated antiaging and antineurodegenerative effects include neurogenesis, autophagy stimulation, and antiapoptotic, antioxidant and anti-inflammatory activities (Choi et al., 2021).

### American ginseng

American ginseng is the dried root of *Panax quinquefolium* L. Cereboost™, an American ginseng extract that contains a high concentration of ginsenoside Rb1, is a well-known component to improve human cognitive function. Cereboost™ has shown AChE inhibitory activity in an AD mouse model (male Institute of Cancer Research (ICR) mice receiving Aβ_1-42_ lateral ventricle injection). Cereboost™ increases the level of AChE in the brain and reduces the level of Aβ_1-42_ by inhibiting AChE while improving the cognitive performance of mice (Shin et al., 2016). *In vitro*, Cereboost™ increases ChAT expression and reduces Aβ1-42 cytotoxicity. The same results were observed in other studies of AD mouse models (scopolamine-injected ICR mice).

### Dendrobium

Dendrobium is the fresh or dry stem of the cultivated product of *Dendrobium nobile* Lindi., *Dendrobium huoshanense* C. Z. Tang et S. J. Cheng, *Dendrobium chrysotoxum* Lindi. or *Dendrobium fimbriatum* Hook. and its similar species. Dendrobium is a traditional Chinese herbal medicine and is slightly cold but sweet in taste. The active ingredient *Dendrobium nobile* total alkaloids (DNLA) is the main active component of Dendrobium. DNLA has antiaging and memory-improving effects. DNLA may reduce the deposition of Aβ in the hippocampus by inhibiting BACE1 protein expression (Zhang M. et al., 2016).DNLA has antiaging, longevity and immunomodulatory effects in animals. The mechanisms by which DNLA improves cognitive dysfunction in animal models of AD may be related to improving the production of extracellular amyloid plaques, regulating tau protein hyperphosphorylation, inhibiting neuroinflammation and neuronal apoptosis, activating autophagy, and enhancing synaptic connections (Li D. D. et al., 2022). Numerous studies have demonstrated that DNLA can effectively improve cognitive deficits in animal models of AD induced by SAMP8, βAPP/PS1 and LPS and prevent Aβ-induced synaptic degeneration in cultured hippocampal neurons. DNLA can activate the Wnt/β-catenin signaling pathway, thereby protecting synaptic integrity, rescuing Aβ-mediated synaptic and mitochondrial damage, and inhibiting amyloidosis *in vitro* and *in vivo* (Zhang W. et al., 2022). Studies have found that DNLA can reduce the loss of neurons and Nissl bodies in the hippocampus and cortex, improve the expansion and swelling of the ER in hippocampal neurons, and reduce the hyperphosphorylation of tau protein, thereby improving learning and memory impairment (Liu et al., 2020). In addition, DNLA can significantly improve spatial learning and memory ability, increase the number of neurons and Nissl bodies, improve neuronal hypertrophy and microstructure, and increase the number of synapses in neurons in AD mice induced by Aβ25-35 (Nie et al., 2016).

Pretreatment with Dendrobium officinale polysaccharide (DOP) contributes to the transformation of BV2 cells from a proinflammatory phenotype to an anti-inflammatory phenotype and enhances the Aβ clearance rate in response to Aβ damage. DOP significantly attenuated cognitive decline in SAMP8 mice. DOP also inhibited the increased activation of hippocampal microglia in SAMP8 mice by downregulating IL-1β, TNF-α, and IL-6 and upregulating IL-10, neprilysin (NEP) and insulin-degrading enzyme (IDE). The accumulation of Aβ42 and p-tau protein in the hippocampus of SAMP8 mice was also reduced, indicating that Dendrobium may provide neuroprotection against AD-related cognitive impairment by regulating the activation of microglia (Feng et al., 2019).

### Polygonum multiflorum

Polygonum multiflorum is the dried root tuber of Polygonum multiflorum Thunb. Polygonum multiflorum is slightly warm in nature and bitter, sweet and astringent in taste, and it has the effect of nourishing essence and blood. Among the components of Polygonum multiflorum, stilbene glycoside (TSG) is the main water-soluble component, and TSG has the functions of nerve protection, antiaging effects and blood vessel relaxation. After the administration of TSG to SAMP8 mice, the expression of the APP and PS1 genes in the hippocampus was significantly reduced, indicating that TSG may prevent and treat AD by downregulating the expression of PS1 (Li et al., 2013). Long-term administration of TSG to AD model mice can reduce the expression of BACE1 and PS1, which can improve the learning, memory and cognition of mice by inhibiting the activity of γ-secretase and reducing the cleavage of Aβ (Chen, 2017).

### Coptis chinensis

Coptis chinensis is the dry rhizome of Coptis chinensis Franch., Coptis deltoidea C. Y. Cheng et Hsiao or Coptisteeta Wall. Berberine is a natural isoquinoline alkaloid extracted from Coptis chinensis. Berberine has shown good results in the treatment of AD, and it can treat neurodegenerative problems as well as neuroinflammation. Berberine is an effective botanical ingredient in the treatment of various neurodegenerative diseases, such as AD. Berberine confines extracellular amyloid plaques and intracellular NFTs. Berberine also has lipid-lowering and hypoglycemic abilities and can inhibit oxidative stress and neuroinflammation in AD; therefore, berberine might be used as a protective agent against atherosclerosis and AD.

AD is a neurodegenerative disease characterized by Aβ plaques, NFTs and neuronal cell death. Active Aβ accumulation accelerates senile plaque formation and interferes with ER function. Changes induced by Aβ accumulation stimulate the unfolded protein response (UPR), which can trigger neuronal apoptosis. The activation of the protein kinase RNA-like endoplasmic reticulum kinase (PERK) is stress-dependent and increases the phosphorylation of eukaryotic translation initiation factor-2α (eIF2α). eIF2α promotes the synthesis of BACE1, which in turn promotes Aβ production and subsequent neuronal apoptosis. Liang et al. (2021) found that berberine treatment inhibited the translation of BACE1 mediated by PERK/eIF2α signaling, thereby reducing Aβ production and resulting in neuronal apoptosis. In addition, berberine may have neuroprotective effects by reducing ER stress and oxidative stress, demonstrating the potential of berberine in the treatment of AD.

In 3×Tg-AD model mice, berberine inhibits Aβ production, attenuates tau hyperphosphorylation, and significantly improves spatial learning and memory retention. In addition, berberine reduces tau levels through the autophagy pathway. Berberine enhances autophagic activity through the PI3K/beclin-1 pathway, thereby promoting autophagic clearance of tau. Berberine can alleviate cognitive decline by simultaneously targeting tau hyperphosphorylation and autophagic clearance of tau in AD mice (Chen Y. et al., 2020). In an AD and type 2 diabetes mellitus (T2DM) combined rat model, berberine alleviated the memory impairment of model rats; restored nerve cell arrangement disorder and neuron damage; improved terminal deoxynucleotidyl transferase-mediated dUTP nick end labeling (TUNEL)-positive cells; and reversed the increase in fasting blood glucose, TG, TC and glycosylated serum protein. In addition, berberine could protect neurons by inhibiting the ER stress pathway and thereby may have some beneficial action in the prevention and treatment of AD and T2DM (Xuan et al., 2020).

Berberine promoted the formation of cerebral microvessels. The new blood vessels promoted by berberine had a complete structure and function, promoted the recovery of CBF, and had neuroprotective effects on 3×Tg-AD mice (Ye et al., 2021).

Alzheimer’s-like disease was orally induced in rats with a mixture of aluminum, cadmium, and fluoride for 3 months, followed by treatment with berberine for another month. Berberine significantly improved cognitive behavior in the MWM test and protected against heavy-metal-induced memory impairment. Berberine inhibited AChE, COX-2 and TACE; downregulated the expression of AChE in brain tissue; and inhibited AChE activity. In addition, berberine induced the production of the antioxidant Aβ40 and inhibited the formation of Aβ42, which is responsible for the aggregation of Aβ plaques (Hussien et al., 2018). In recent years, various mechanisms of the actions of berberine on AD have been gradually discovered and reported, including the inhibition of Aβ deposition, the inhibition of cholinesterase and monoamine oxidase, and the delay of the occurrence of neuroinflammation and oxidative stress (Gao and Chu, 2017). In AD model rats injected with D-gal combined with Aβ25-35, berberine improved learning and memory, and its mechanism was related to the reduction in GSK3β and p-tau protein expression in the rat hippocampus (Zhou L. et al., 2020).

### Ligusticum chuanxiong

Ligusticum chuanxiong: The dried rhizome of Ligusticum chuanxiong Hort., Ligusticum chuanxiong is warm in nature and sweet in taste, and it has the effects of promoting blood circulation, activating qi, dispelling rheumatism and relieving pain. Ligusticum chuanxiong mainly contains alkaloids, volatile oils, phenols and visfatin. Ligustrazine is an alkaloid extracted from Ligusticum chuanxiong. Ligustrazine dilates blood vessels, improves cerebral ischemia and protects neurons, and it has been clinically used to treat cerebrovascular diseases. A study found that the number of NEP-positive cells in the hippocampus of D-gal/AlCl3 mice in the ligustrazine group was significantly higher than that of mice in the control group, which indicated that ligustrazine could increase the expression of NEP, promote the clearance of Aβ, and thus play a role in the prevention and treatment of AD (Liu Z. et al., 2016).

Hyperhomocysteinemia (Hhcy) is an independent risk factor for AD. Hhcy can cause memory impairment through AD-like tau and Aβ pathology in the hippocampus. Lustrazine significantly attenuated Hhcy-induced tau hyperphosphorylation and Aβ accumulation in the Hhcy rat model (Zhang Q. et al., 2021).

In 3xTg-AD and APP/PS1-AD mice, ligustrazine significantly improved the learning and memory ability of these two kinds of AD transgenic mice, reduced Aβ and p-tau levels, altered the seahorse proteome and reduced AD pathology (Huang et al., 2021).

Ligustrazine inhibited the molecular events of mitochondrial dysfunction and mitochondrial apoptosis by enhancing Nrf2/GCLc/GSH and inhibiting the HIF1α/NOX2/ROS pathway, thereby protecting against CoCl2-induced neurotoxicity in PC12 cells and rats (Guan et al., 2015).

### Huperzia serrata

*Huperzia serrata*: The whole herbs of Huperziaserrata (Thunb.) Trev. [Lycopodiumserratum Thunb.]. HupA is a huperzine alkaloid derived from the Chinese herbal medicine *Huperzia serrata*. HupA has multiple targets and can competitively inhibit AChE activity and reduce neuronal cell death. In addition, HupA was shown to protect neuronal cells against the oxidative stress induced by Aβ, a protein kinase C inhibitor, and free-radical-induced apoptosis. Along with Ach, butyrylcholinesterase is also a part of the cholinesterase system and is expressed in neurons and glial cells. Clinical studies have found that butyrylcholinesterase also plays an important role in senile plaques and NFTs in AD patients. A double-blind study involving 50 AD patients and 50 healthy individuals found significant improvements in cognition and task switching ability after HupA treatment (Gul et al., 2019). Studies have shown that huperzine A is superior to placebo in improving the memory quotient and the score of MMSE in patients with mild cognitive impairment and has a low incidence of adverse reactions, which has good clinical safety. Similarly, huperzine A could significantly improve the memory, cognitive function and self-care ability of vascular dementia patients, which was statistically significant compared with the control group and was less affected by publication bias. Huperzine A has side effects, but the symptoms are mild, which generally does not affect the continued administration of the drug (Hu et al., 2018).

In addition to the symptomatic cognitive-enhancing effects through the inhibition of AChE, a recent study suggested that HupA had a “noncholinergic” effect on AD. HupA activated α-secretase cleavage and downregulated β/γ-secretase, enhanced BDNF/TrkB signaling and the PI3K/Akt and PI3K/TrkB/mTOR pathways, and combated Aβ-induced oxidative damage and mitochondrial dysfunction while reducing IL-1β, IL-6, TNF-α and NF-kB signaling to protect neuronal function. Moreover, HupA could restore Fe^2+^ homeostasis in the brain. These findings further support that HupA may significantly slow the neuronal death process (Friedli and Inestrosa, 2021).

A clinical trial showed that *Huperzia serrata* extract could also improve memory functions, reduce visual hallucinations and night talking, and ameliorate activities of daily living, verbal communication, scowling and the disappearance of unhygienic behavior (Tabira and Kawamura, 2018).

### Polygonum cuspidatum

*Polygonum cuspidatu*m: The dried rhizome and root of *Polygonum cuspidatum* Sieb. et Zucc. Physion8-O-β-glucopyranoside (PSG) isolated from Polygonum cuspidatum can significantly enhance the learning and memory ability of rats with Aβ1-40-induced dementia through lateral ventricle injection, which may be related to increasing the levels of Ach, 5-HT, NE, and DA in the hippocampus; reducing the content of Aβ; and upregulating the expression of drebrin (Xu et al., 2015). The alcohol extract of Polygonum cuspidatum may reduce the Aβ level in the brains of AD model mice through the AMPK/PGC-1α/BDNF/TRKB signaling pathway, thereby improving their learning and memory ability (Zhang et al., 2019).

### Gardenia

Gardenia is the dry ripe fruit of *Gardenia jasminoides* Ellis. Geniposide is an iridoid glycoside that is mainly found in the dry and ripe fruit of *Gardenia jasminoides* Ellis. Pharmaceutical studies have confirmed that in addition to antipyretic, analgesic and anti-inflammatory effects, geniposide can also have some effect on neurodegenerative diseases, such as PD. Possible molecular mechanisms by which geniposide protects the brain from AD pathological damage are reducing amyloid plaques, inhibiting tau phosphorylation, preventing memory impairment and synaptic loss, reducing oxidative stress and chronic inflammatory responses, and promoting neurite outgrowth through the glucagon-like peptide-1 receptor (GLP-1R) signaling pathway (Liu et al., 2015). In addition, geniposide is a water-soluble small-molecule drug that can pass through the BBB with a high permeability; therefore, theoretically, geniposide has a better effect on CNS diseases. Geniposide has a protective effect on Aβ25-35-induced neuronal damage and significantly improves cognitive and behavioral abilities in AD rats. The mechanism of action of geniposide may be related to the activation of the ERK1/2-Nrf2 signaling pathway (Xiang et al., 2018).

Gardenia essential oil aromatherapy can reduce the body weight and anxiety of APP/PS1 mice, increase their exploration ability, inhibit fat accumulation, improve APP/PS1, and improve exercise tolerance and hindlimb blood flow velocity in rats. (Chen Z. et al., 2020)

Geniposide has been proven to significantly reduce cerebral infarct size and alleviate neuronal damage and necrosis by inhibiting inflammatory signals, including NLRP3, TNF-α, IL-6, and IL-1β. Activation of the PI3K/Akt and Wnt/catenin pathways is also involved in neuronal protection. In the treatment of AD, geniposide increases autophagy and inhibits apoptosis by regulating the function of mTOR. Geniposide has also been shown to be a GLP-1R agonist that reduces amyloid plaques and inhibits oxidative stress to alleviate memory impairment and synaptic loss (Zhang W. et al., 2021).

### Polygala

Polygala is the dried root of Polygalactaceae Polygalatenuifolia Willd. or Polygalasibirica L. Polygala is a drug used to improve memory and cognitive function. Polygala has the effects of calming nerves, benefiting intelligence, relieving depression and so on. Polygala mainly contains triterpenoid saponins, ketones, oligosaccharide esters and other compounds. Polygala and its components have anti-Aβ, tau protein regulatory, anti-inflammatory, antioxidant, and antiapoptotic effects. Polygala can inhibit the development of brain neuron degeneration in multiple ways and may be effective in the prevention and treatment of AD (Li X. et al., 2022). A study found that in an AD model induced by Aβ1-40, Polygala significantly protected against the reduction in the number of neurons, plaque deposition and neuronal apoptosis in the brain tissue by improving the function of the ubiquitin–proteasome pathway in the hippocampus, counteracting Aβ neurotoxicity and improving AD memory impairment (Sun et al., 2013). Polygala can increase the expression of O-linked β-N-acetylglucosamine (O-GlcNAc) to a certain extent, regulate the balance between protein kinase and phosphatase in an AD rat model, and return the phosphorylation of tau protein to a normal physiological state (Chen et al., 2013). Polgala saponins can reduce the expression of Taup-Ser2 protein and PKA in AD model rats, increase the expression of PP2A protein, reduce the hyperphosphorylation of the Ser396 site of tau protein in brain neurons of AD rats, and protect nerve cells from Aβ1-40 toxicity (Chen et al., 2015).Polygala powder can inhibit the phosphorylation of tau protein in the hippocampal CA1 region of model rats in which AD is induced by Aβ1-40, and its mechanism is related to the regulation of the PI3K/AKT/GSK-3β signaling pathway (Xie P. et al., 2020). Polygala saponins can upregulate the expression of PSD-95 in the hippocampus of AD mice and improve cognitive impairment, which may be achieved by reducing Aβ deposition and the hyperphosphorylation of tau protein (Wang Z. et al., 2020). It has also been confirmed that polygala saponins have potential benefits in the treatment of learning and memory deficits in APP/PS1 transgenic AD mice, and their effects may be related to the reversal of AD pathology-induced neuronal apoptosis (Wang L. et al., 2019).

In 5XFAD transgenic AD model mice, Polygala radix extract can inhibit neuronal endocytosis and prevent axonal degeneration and memory impairment; in cultured neurons, Polygala radix extract can prevent Aβ-induced collapse of axonal growth cones and axonal growth cone atrophy and prevent the Aβ-induced endocytosis of cultured neuronal growth cones (Kuboyama et al., 2017).

### Bergamot

Bergamot is the dried fruit of Citrusmedica L. var. sarcodactylis Swingle. The application of aromatherapy in AD has become a research hotspot among scholars. Bergamot essential oil (BEO), one of the most common essential oils, is composed of volatile aromatic terpenes and oxygenated derivatives such as linalool, and previous studies have demonstrated the neuroprotective and anti-inflammatory properties of BEO (Scuteri et al., 2019).The inhalation of BEO for 21 days alleviated the cognitive behavioral impairment of AD mice and reduced Aβ content and AChE activity in the hippocampus and cortex in D-gal- and aluminum-trichloride-treated AD mice (Hu et al., 2022).

### Rhodiola

Rhodiola is the dried root and rhizome of *Rhodiola rosea* Rhodiola (Hook. f. et Thoms.) H. ohba, a plant of the Crassulaceae family. With the continuous development of and improvements in scientific research technology, a large number of studies have been carried out on the chemical constituents of Rhodiola. The main pharmacologically active components isolated from various Rhodiola species are salidroside (Sal) and its aglycone (tyrosol), rhodopsin, pyridine and rhodiola. Among them, Sal is a phenolic glycoside compound that is the main active ingredient extracted from Rhodiola. This can effectively reduce the deposition of Aβ1-42 and neuroinflammation in the brains of AD mice and improve cognitive decline during aging, thereby exerting neuronal protection. In an *in vitro* model of PC12 cell injury induced by D-gal and nigericin, Sal reversed TLR4, MyD88, NF-κB, P-NF-κB, NLRP3, and ASC and cleaved Caspase-1 and increased the protein expression of cleaved GSDMD, IL-1β and IL-18. The mechanism may be through inhibition of the TLR4/NF-κB/NLRP3/Caspase-1 signaling pathway. In animal models of AD induced by Aβ1-42 and D-gal/AlCl3, Sal can improve AD by inhibiting pyroptosis, reducing tau phosphorylation, and reducing Aβ accumulation (Cai et al., 2021).

Sal effectively attenuates hippocampal-dependent memory impairment in SAMP8 mice, possibly by modulating the gut-microbiota-brain axis and inflammation in the peripheral circulation and CNS. The relevant mechanisms of action include significantly reducing toxic Aβ1-42 deposition; activating microglia in the brain; reducing the levels of the proinflammatory factors IL-1β, IL-6 and TNF-α; improving gut barrier integrity and the gut microbiota; reversing the ratio of *Bacteroides* to Firmicutes; eliminating Clostridium and Streptococcus, which may be associated with cognitive deficit; and reducing the levels of proinflammatory cytokines in the peripheral circulation, especially IL-1α, IL-6, IL-17A, and IL-12 (Xie Z. et al., 2020).

Sal improved the motor activity of APP/PS1; reduced soluble and insoluble Aβ levels; and increased the expression of PSD95, NMDAR1 and calmodulin-dependent protein kinase II. Phosphatidyl inosine PI3K/Akt/mTOR signaling was upregulated. These results suggest that Sal may protect APP/PS1 mice from neuronal synapse damage (Wang H. et al., 2020).

### Acorus tatarinowii

*Acorus tatarinowii* is the dried rhizome of *Acorus tatarinowii* Schott, a plant of the Araceae family. According to reports, *Acorus tatarinowii* can repair the synaptic structure and improve learning and memory by inhibiting the overexpression of APP, reducing the production of Aβ, and reducing Aβ damage to neurons and synaptic structures (Zhang et al., 2014).

β-Asarone (BAS) is the main component of *Acorus tatarinowii*, and it has an important effect on the CNS. BAS can inhibit Aβ, which may be achieved by promoting autophagy in AD cell models (Wang N. et al., 2019). The neuroprotective effects of BAS were investigated in the APP/PS1 double-transgenic mouse model and NG108 cells, and it was found that BAS antagonized Aβ neurotoxicity *in vivo*, improved the learning and memory abilities of APP/PS1 mice, and increased synaptophysin and glutamate receptor 1 expression *in vitro* and *in vivo* (Liu S. J. et al., 2016). The study found that after 30 days of BAS intervention, BAS effectively promoted the mitotic phagocytosis mediated by PTEN-induced kinase 1 and Parkin RBRE3 ubiquitin protein ligase and improved the learning and memory ability of rats in which AD was induced by Aβ1-42 (Han et al., 2020). BAS reduced neuronal apoptosis and Aβ deposition in the cerebral cortex and hippocampus and downregulated Aβ1-42 levels, improving memory deficits in APP/PS1 double-transgenic AD model mice (Yang et al., 2016).

BAS reduced the number of senile plaques and autophagosomes in the hippocampus of APP/PS1 double-transgenic mice, and it reduced the expression of Aβ40, Aβ42, and APP, which have neuroprotective effects and inhibit autophagy (Deng et al., 2020). BAS reduced the levels of AChE and Aβ42 and inhibited Beclin-1-dependent autophagy through the PI3K/Akt/mTOR pathway, thereby improving the learning and memory abilities of APP/PS1 transgenic mice (Deng et al., 2016).

In Aβ-stimulated PC12 cells, BAS improved cell viability and reduced cell damage and apoptosis. BAS also reduced ROS and MDA levels; increased SOD, CAT and GSH-PX levels; improved mitochondrial membrane potential; and exerted neuroprotective effects by regulating the P13K/Akt/Nrf2 signaling pathway (Meng et al., 2021).

### Gastrodia elata

*Gastrodia elata* is the dried tuber of *Gastrodia elata* Bl. *Gastrodia elata* is a commonly used TCM for the treatment of cerebrovascular diseases, and *Gastrodia elata* extract or its active components have physiological and health-promoting functions, including antitumor, memory-improving, and neuroprotective activities. *Gastrodia elata* may prevent AD by modulating the proteolytic process of APP, thereby driving nonamyloidogenic pathways, and *Gastrodia elata* may be a promising adjuvant therapy for AD (Heese, 2020). In an AD rat model established by bilateral hippocampal injection of Aβ25-35, after 52 days of *Gastrodia elata* intervention, the spatial memory of the model rats was significantly improved, and the number of amyloid deposits in the rat hippocampus was significantly reduced; the medial septal nucleus (medial septum, MS) and the expression of ChAT in the hippocampus were significantly increased; and the activity of AChE in the prefrontal cortex, medial septum, and hippocampus was significantly decreased, indicating that long-term administration of *Gastrodia elata* has therapeutic potential for AD (Huang et al., 2013). In scopolamine-induced memory impairment, rat model studies have demonstrated that *Gastrodia elata* may potentially ameliorate the learning and memory deficits and/or cognitive impairment caused by neuronal cell death (Park et al., 2015).

Gastrodin is a phenolic glycoside and the main bioactive component of *Gastrodia elata*. The pharmacological properties of gastrodin have been extensively studied since its identification in 1978. Gastrodin has a wide range of beneficial effects on CNS diseases, and its mechanisms of action include the modulation of neurotransmitters, antioxidants, and anti-inflammatory factors; the inhibition of microglial activation; the modulation of mitochondrial cascades; and the upregulation of neurotrophins (Liu Y. et al., 2018). In an AD mouse model (Tg2576 mice), gastrodin significantly attenuated Aβ deposition and glial cell activation in the brains of these transgenic mice, suggesting that gastrodin can exert neuroprotective effects through anti-inflammatory and antiamyloidogenic effects (Hu et al., 2014). In a rat model of VD induced by bilateral common carotid artery occlusion, gastrodin treatment inhibited the deposition of Aβ plaques by reducing the level of p-tau in the hippocampus, thereby improving cognitive deficits in VD rats (Shi et al., 2020).

### Lycium barbarum

*Lycium barbarum* is the dry ripe fruit of *Lycium barbarum* L., a plant of the Solanaceae family. *Lycium barbarum* has historically been used as a natural antioxidant and antiaging product.

Multiple *in vitro* and *in vivo* aging models were used to demonstrate the ability of *Lycium barbarum* polysaccharide (LBP) to reduce oxidative stress, restore mitochondrial function, alleviate DNA damage and prevent cellular aging. The findings from these studies suggest that LBP attenuates the accumulation of senescent cells and suppresses senescence-associated secretory phenotypes by reducing the levels of proinflammatory cytokines such as interleukin-1β and matrix-degrading enzymes such as MMP-1 and MMP-13 (Ni et al., 2021). LBP has the ability to protect neurons from Aβ peptide neurotoxicity. LBP has neuroprotective effects on cortical neurons exposed to homocysteine (Ho et al., 2010). LBP can reduce Aβ levels and improve cognitive function in APP/PS1 transgenic mice. LBP1 enhances neurogenesis and restores synaptic dysfunction in the hippocampal CA3-CA1 pathway, as shown by BrdU/NeuN double labeling, and *in vitro* cellular analysis findings suggest that LBP1 may affect Aβ processing (Zhou Y. et al., 2020). Recently, studies have demonstrated that in 3xTg-AD mice, *Lycium barbarum* extract can maintain synaptic stability in the retina by reducing oxidative stress and intracellular calcium influx. Therefore, *Lycium barbarum* extract may act as a neuroprotective agent for AD by preserving synapses (Liu et al., 2021). The function and molecular mechanism of *Lycium barbarum* extract in AD were investigated in transgenic *C. elegans* strain CL 2006. *Lycium barbarum* extract had a high antioxidant capacity and effectively delayed Aβ-induced paralysis in the CL2006 strain. *Lycium barbarum* extract inhibits the production of excessive ROS by inducing the protein skinhead-1 (SKN-1)-mediated antioxidant system, thereby inhibiting the production of Aβ and inhibiting mitochondrial damage. Importantly, LBP reduces Aβ levels by inducing follitropin receptor-mediated activation of the mitochondrial UPR (Meng et al., 2022). Another study found that wolfberry extract treatment significantly improved learning and memory deficits in 3xTg-AD mice, significantly reduced Aβ deposition in 3xTg-AD mice, increased NeuN-positive cells, and upregulated the expression of BDNF and TrkB (Ye et al., 2015). In APP/PS1 mice, fruitless *Lycium barbarum* sprout extract also shows potential for the treatment of AD; it could significantly inhibit Aβ fibrillation, decompose the formed Aβ fibers, reduce Aβ oligomer levels and Aβ-induced neurotoxicity, and attenuate oxidative stress *in vitro* (Liu S. Y. et al., 2018).

### Cistanche deserticola

*Cistanche deserticola* is the dry, scaly, leafy, succulent stem of *Cistanche deserticola* Y. C. Ma or *Cistanche tubulosa* (Schenk) Wight.

Long-term administration of D-gal to mice results in cognitive decline, gut microbial dysbiosis, peripheral inflammation, and oxidative stress. In this model of age-related cognitive decline, *Cistanche deserticola* polysaccharide (CDPS) improved cognitive function in D-gal-treated mice by restoring gut microbial homeostasis, thereby reducing oxidative stress and peripheral inflammation. The beneficial effects of CDPS in these aging model mice were abrogated by immunosuppression with antibiotics or cyclophosphamide. Serum metabolomic analysis showed that the levels of creatinine, valine, L-methionine, o-toluidine, N-ethylaniline, uric acid, and proline were altered in aging model mice, but CDPS could restore them. CDPS improves cognitive function by restoring the homeostasis of the gut-microbiota-brain axis, thereby alleviating amino acid imbalance, peripheral inflammation, and oxidative stress in D-gal-treated mice. Thus, CDPS shows therapeutic potential for patients with memory and learning disabilities, especially those associated with gut microbiome dysbiosis (Gao et al., 2021).

Acteoside (verbenoside), one of the main active phenethyl glycosides of *Cistanche deserticola*, is known to have antioxidant and neuroprotective activities, and herbs containing acteoside can be used to enhance memory. Acteoside shortened the latency and decreased the number of errors in a step test; significantly increased hippocampal neurons and Nissl bodies; and reduced nitric oxide content, nitric oxide synthase activity and caspase-3 protein expression in D-gal- and aluminum-trichloride-treated mice (Peng et al., 2015).

## Conclusion

In summary, the pathogenesis of AD has not been systematically clarified thus far, and the possible pathogenic factors of AD are diverse. In this review, it has been shown that dialectical treatment of AD with TCMs with complex and diverse components has achieved unexpected special curative effects. However, the following aspects need to be further improved: TCMs, especially TCM compounds, have complex components. At present, there is no systematic explanation of which drugs or components are working, and it is more difficult to clarify their action mechanisms and compatibility laws in modern terms. Second, there are some clinical data from observational studies and reference-controlled or add-on treatment studies, and only for a few treatments has clinical efficacy been demonstrated by randomized, placebo-controlled trials. Recent research has mostly been in the form of experimental research or sporadic clinical research reports, and the clinical efficacy indicators of TCMs need to be improved. There are great differences between TCM and Western medicine in diagnosis, the choice of therapeutic drugs and the determination of curative effect. How to introduce effective ancient and tested prescriptions of TCM for the clinical treatment of AD to Western medical workers is a difficult problem that urgently needs to be overcome. Only by finding the ideal combination point of the two medical systems can it be possible for the systems to work together to overcome the difficulties of AD.
